# Influence of molecular designs on polaronic and vibrational transitions in a conjugated push-pull copolymer

**DOI:** 10.1038/srep35096

**Published:** 2016-10-12

**Authors:** Christoph Cobet, Jacek Gasiorowski, Reghu Menon, Kurt Hingerl, Stefanie Schlager, Matthew S. White, Helmut Neugebauer, N. Serdar Sariciftci, Philipp Stadler

**Affiliations:** 1 Center for Surface- and Nanoanalytics, Johannes Kepler University of Linz, A-4040 Linz, Austria; 2 Physics Department, Technical University of Chemnitz, 09107 Chemnitz, Germany; 3 Department of Physics, Indian Institute of Science, Bangalore 560012, India; 4 Institute of Physical Chemistry, Johannes Kepler University Linz, Altenbergerstr. 69, 4040 Linz, Austria; 5 Department of Physics, University of Vermont, Cook Building, 82 University Place, University of Vermont Burlington, VT 05405-0125, USA

## Abstract

Electron-phonon interactions of free charge-carriers in doped pi-conjugated polymers are conceptually described by 1-dimensional (1D) delocalization. Thereby, polaronic transitions fit the 1D-Froehlich model in quasi-confined chains. However, recent developments in conjugated polymers have diversified the backbones to become elaborate heterocylcic macromolecules. Their complexity makes it difficult to investigate the electron-phonon coupling. In this work we resolve the electron-phonon interactions in the ground and doped state in a complex push-pull polymer. We focus on the polaronic transitions using *in-situ* spectroscopy to work out the differences between single-unit and push-pull systems to obtain the desired structural- electronic correlations in the doped state. We apply the classic 1D-Froehlich model to generate optical model fits. Interestingly, we find the 1D-approach in push-pull polarons in agreement to the model, pointing at the strong 1D-character and plain electronic structure of the push-pull structure. In contrast, polarons in the single-unit polymer emerge to a multi- dimensional problem difficult to resolve due to their anisotropy. Thus, we report an enhancement of the 1D-character by the push-pull concept in the doped state - an important view in light of the main purpose of push-pull polymers for photovoltaic devices.

*In-situ* (operando) spectroscopy on free carrier induced absorption in *π*-conjugated polymers displays the pronounced electron-phonon coupling due to *π*-fluctuations. Similar to Raman modes in the ground state, vibronic terms in doped polymers known as infrared-activated vibrations (IRAVs) display symmetry breaking due to presence of charge carriers^1^,^2^,^3^,^4^. Originally, these modes have been described in doped and photoexcited simple conjugated systems such as polyacetylene and polythiophenes, where all CSC, CCC and CCH vibrations can be assigned to the spectral response. Meanwhile, polymers developed to more complicated macromolecular structures, so that one-to-one vibrational assignments become difficult. However, there exists crucial interest to resolve the doped state electronics as it provides an insight to optoelectronic devices during operation^5^,^6^,^7^,^8^,^9^,^10^. In this work we took such an *in-situ* operando view to these electronic transitions, which emerge only in the doped state. Electronically these are polaron transitions, which qualitatively relate to the coupling of phonons and free charge carriers. Fitting of these transitions allows an estimation of the strength of inter- and intramolecular forces and hence provides substantial information about carrier dynamics, which can be directly proved in device-structures. We elucidate these dynamics for the first time in a sophisticated push-pull polymer such as PBDTTT-c both in the material and in a device-related style. Interestingly we find agreement with earlier results on 1D delocalization, which appears specifically enhanced by push-pull molecular structures.

## Results

Typically polarons emerge from electron-phonon interactions. Their binding energy and the intensity of IR absorption due to polaron excitations scales with a dimensionless coupling constant according to Froehlich


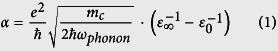


where *m*_*c*_ the effective electron mass and *ω*_*Ph*_ is the LO-phonon frequency. In conjugated polymers polaron absorption depends strongly on dielectric function between *ε*_∞_ and *ε*_0_ (high-frequency electronic and static dielectric constant). The term 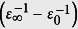 defines the effective dielectric constant related to the lattice polarisability. For a given electronic polarisability *ε*_∞_ maximizes if the measurable static dielectric constant becomes large. Furthermore it is evident that the electron-phonon coupling and thus the intensity of polaronic absorption increase with electron localization i.e. in case of a high effecitve electron mass *m*_*c*_.

In this study we compare regioregular poly-3-hexylthiophene (rr-P3HT) and a prominent push-pull system poly[(4,8-bis-(2-ethylhexyloxy)-benzo(1,2-b:4,5-b’)dithiophene)-2,6-diyl-alt-(4-(2-ethylhexanoyl)-thieno[3,4-b]thiophene-)-2-6-diyl] (PBDTTT-c)^11^,^12^. The latter reflects exactly recent developments, where intramolecular dipoles red-shift the band gap to explore infrared (IR) activity. In PBDTTT-c, it is benzodithiophene and thienothiophene, which serve as sequential donor-acceptor pair^13^,^14^,^15^,^16^,^17^,^18^. The IR-activity in push-pull polymers usually pays off by an extraordinary molecular complexity - the number of carbon atoms per monomer increased by a factor of 4 from P3HT to PBDTTT-c. Typically, push-pull polymers afford also an elaborate side-chain engineering to obtain solubility^19^,^20^,^21^,^22^,^23^. Taking PBDTTT-c as an example, little is know about the influence of such complexity to the polaron levels. Therefore we pursue an operando spectroscopic study to achieve this insight, in particular on polaron dynamics^24^,^25^,^26^,^27^,^28^,^29^,^30^.

On basis of ground-state and doped-state data we create optical models to visualise polaron differences. Generic oscillators are useful here to assemble these corresponding delta-functions with strong broadening effects (Lorentz-type or Gaussian-type). This allows us to conclude on the molecular interactions dominating the solid-state system^31^,^32^. Interestingly we find the complex push-pull polymer to suppress intermolecular forces consequently fitting well 1D-delocalization. This is opposite to highly-crystalline rr-P3HT, where the wave functions spread into inter- and intramolecular identities with diverging intensity. Our results show, that the push-pull concept strengthens the 1D-character - it can drive a conjugated polymer with complex molecular structure towards a simple advantageous optoelectronic structural fingerprint.

**Figure 1:**
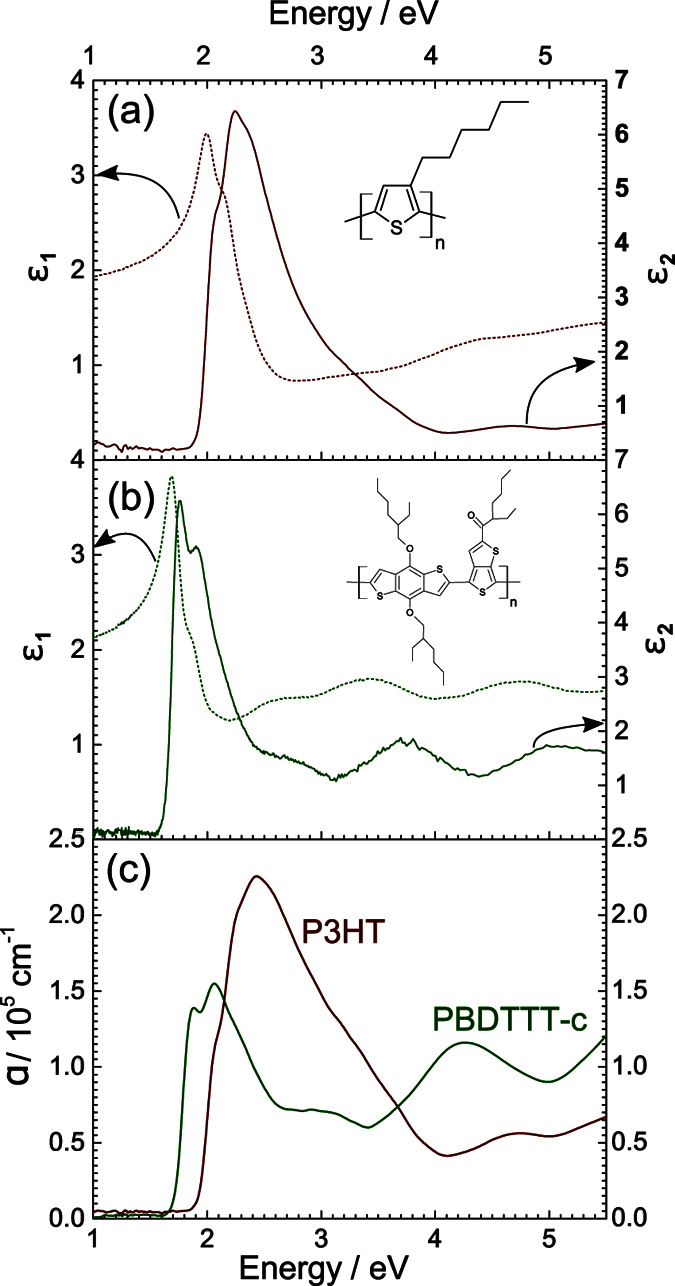
Ellipsometric spectra of ground state rr-P3HT and PBDTTT-c including molecular structure. (**a**,**b**) The dielectric functions *ε*_1_, *ε*_2_ correspond to the real (left, *ε*_1_) and imaginary (right, *ε*_2_) part as indicated in the graphs. In (**c**) we plot the absorption coefficient of both polymers.

The experimental optical model for the pristine polymers (Fig. 1) are shown in the dielectric functions (and the absorption coefficient *α*) for PBDTTT-c and rr-P3HT, respectively. The latter (Fig. 1a) has maxima at 2.1 and 2.3 eV (imaginary part left scale, *ε*_2_). The feature splitting is a signature of intra- and intermolecular alignment and therewith *π* − *π* stacking^33^: This is true for the absorption features between 2 and 4 eV. In detail the shoulder at 2.07 eV is attributed to interchain-delocalized exciton, while the following peaks at 2.27 eV and 2.49 eV are phonon replica of the exciton. The broad absorption shoulder at 2.6 eV refers to the screened *π* - π* transition^34^. The onset of the absorption is found at 1.9 eV^35^. The dispersion - consistent with absorption - is reflected in the plot of the real part *ε*_1_ having two strong variations that are connected with two oscillators with maxima at 1.95 and 2.1 eV. All mentioned assignments fit measurements on films and dilluted rr-P3HT. Thus we derive a consistent value for the average LO-phonon frequency *ω*_*Ph*_ at 190 to 200 meV. PBDTTT-c shows different absorption features: Uniting donor (thieno[2,3-b]thiophene) and acceptor (benzodithiophene with alkoxy-side-chains) red-shift the absorption onset to 1.55 eV. The dielectric functions is presented in Fig. 1b with two sharp peaks at 1.75 eV (*π*-π* transition) and 1.9 eV. Unlike rr-P3HT, this splitting is not originated from *π*-*π* - stacking but rather a fingerprint of the push-pull character. In addition to the dielectric function we include the absorption coefficients *α* for both polymers (Fig. 1c). In rr-P3HT, a different spectral shape of *α* as compared to *ε*_2_ is apparent - it originates from the important role of the refractive index n_i_ in thin films. The absorption coefficient has a broad peak with maximum at 2.4 eV, which is a result of dispersion from three oscillators describing the optical transitions. Differently, the absorption coefficient for PBDTTT-c correlates better to the *ε*_1_-function.

**Figure 2:**
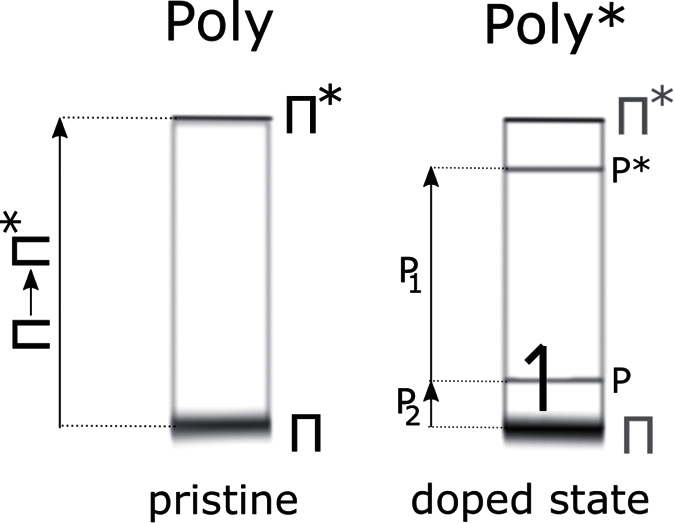
Schematic illustration of the ground state and doped state in a *π*-conjugated polymer. The characteristic in-gap polaron transitions P_1,2_ are indicated.

For conducting *in-situ* spectroscopy on the doped-state, we create a long-lived (persistent) species, which allows elucidation by variable angle spectroscopic ellipsometry (VASE) and attenuated total reflection (ATR) FTIR. Persistency is achieved by iodine-doping rr-P3HT and PBDTTT-c while recording spectra. Alternatively, dynamic doping uses the photo-excitation of the polymer in presence of electron acceptor phenyl-C61-butyric acid methyl ester (PCBM). The latter method mimics the charge-separation processes in an organic photovoltaic cell (bulk-heterojunction). We denote that photo-induced *in-situ* absorption (PIA) measurements are presented for ATR-FTIR in the mid-IR^36^,^37^. Combined with persistent doping we obtain a complete survey covering the spectral region between 5.5 and 0.075 eV exactly where vibronic transitions (IRAVs), polaron transitions (P_1,2_ and ground state transitions occur (Fig. 2).

**Figure 3:**
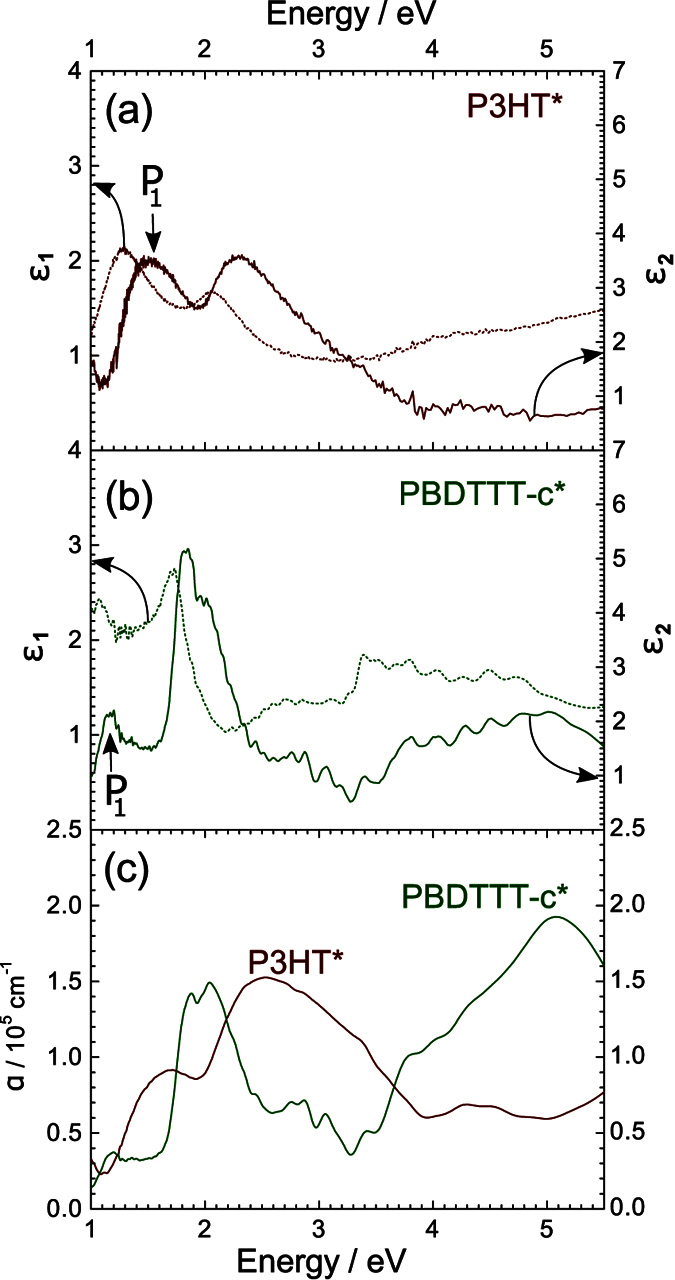
Ellipsometric doped-state spectra on persistently doped rr-P3HT and PBDTTT-c. In (**a**,**b**) the rise of the P_1_ polarons for both systems (with different intensities) is indicated. The response function of the absorption coefficient in the doped state is also shown in (**c**).

At first we present datasets in the UV-visible and near-IR (5 to 1.0 eV) of doped-state levels introduced by persistent chemical doping (Fig. 3). We show the real (*ε*_1_) and imaginary part (*ε*_2_) of the dielectric function of doped polymers rr-P3HT* and PBDTTT-c*, respectively. For P3HT* *ε*_2_ shows a quenched and broad main absorption peak signal with a maximum at 2.3 eV and a transition betweeen localized polaron state at 1.5 eV, denoted as P_1_. Above 3 eV we cannot observe changes as compared to the ground-state, so we conclude that doping affects mainly *π*-levels (polarons disrupt loosley-bound HOMO and LUMO states). Contributions from iodine are not apparent (rr-P3HT and PBDTTT-c*). The rr-P3HT* real dielectric function *ε*_1_ repeats the ground state oscillator quenching and displays the concomitant formation of an in-gap P_1_ transition at 1.5 eV.

For PBDTTT-c*, we see a similar impact: Quenching in the active absorption region and rise of P_1_. The maxima of PBDTTT-c* are red-shifted as compared to rr-P3HT* due to the lower absorption edge in the ground state. Concretely, P_1_ arises at 1.2 eV (*ε*_2_) (absorption coefficients in Fig. 3c). Between P3HT and P3HT* the decrease of the original absorption feature is significant, which is not the case between PBDTTT-c and PBDTTT-c* - the quantitative changes due to doping are minor, which correlates to the higher oxidation potential of PBDTTT-c.

To probe the low-energy regime with the continuum polaronic excitations (P_2_) and the IRAVs, we change to *in-situ* ATR-FTIR to cover the spectral mid-IR region between 0.6 to 0.075 eV. For P3HT* we see a characteristic broad P_2_ feature above 0.18 eV to 0.19 with a maximum at 0.35 eV. Below 0.18 eV IRAVs emerge characterized by multiple, intense absorption peaks. In parallel, PBDTTT-c* exhibits a broad P_2_ transition with a maximum at 0.39 eV including IRAVs. The latter are broadened as compared to rr-P3HT.

**Figure 4:**
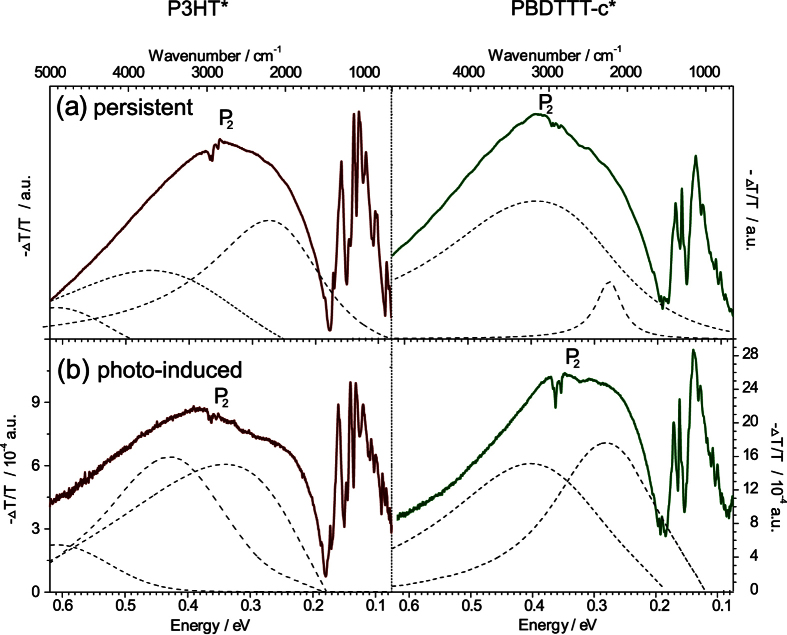
Juxtaposition of persistently- and photodoped *in-situ* spectra by ATR-FTIR exploring P_2_ transitions. In the mid-IR regime rr-P3HT (left) and PBDTTT-c (right) show the broad polaronic transitions (P_2_) and lower energy IRAV modes. We included generic oscillators (dashed lines) from the underlying model fit - their superposition renders the experimental spectra in precision.

In ATR-FTIR, photo-inducted absorption is accessible too. We crosschecked, how doped-state levels are affected by the origin of excitation. In both polymers we cannot report on substantial differences in the spectral response (Fig. 4), unless taking into account quantitative evaluations. Persistent doping with iodide yield quantitative changes, while photo-induced changes are relatively small and, though noise-reduced due to lock-in data acquisition, less precise. Qualitatively, we find the maxima for IRAVs and P_2_ transitions at the same positions with minor deviations as indicated in Fig. 4, independently, which method for excitation has been applied. For comparison, we sum up all data on in-gap states in Table 1.

## Discussion

**Figure 5:**
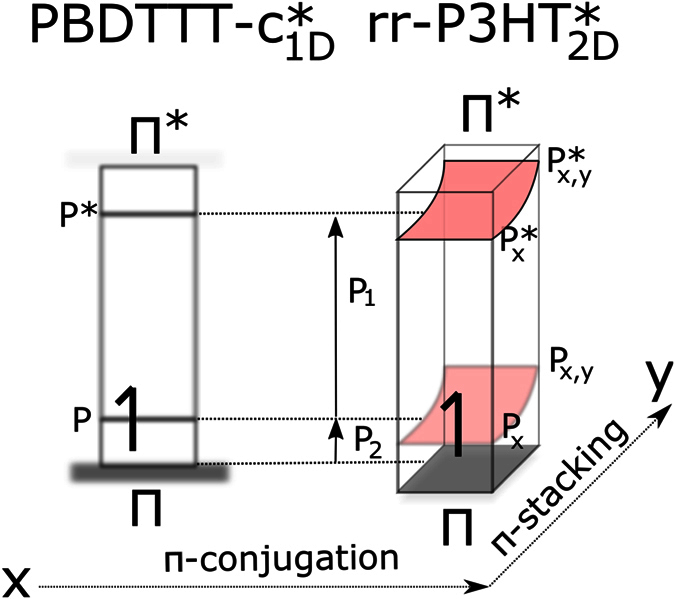
Simplified schematic of 1D and 2D-polarons as found for PBDTTT-c and rr-P3HT. We point at the main finding of *in-situ* spectroscopy that the push-pull system enhances 1D-delocalization, while the single unit system shows a comparatively complex doped-state electronic structure.

The interesting part in evaluation of the presented doped-state spectra affects the differences within the polymer systems. Common rr-P3HT has a simple molecular desing but features a complex physical insight: Our approach fitting spectra with a minimum amount of generic oscillators fails, when we stick to a 1D Froehlich-model (details explained in SI). As already discussed in Fig. 1c, rr-P3HT exhibits strong dispersion effects in the ground state likely originating from the strong *π*-stacking. Therefore the minimum amount of generic oscillators used to reasonably fit the data are 3, which are illustrated in Fig. 4, left. The polaronic absorption lines are then in agreement, if additional intermolecular forces are considered. In the literature, this effect has been also assigned as 2D-polaron^8^,^38^,^39^,^40^ - accordingly we illustrate the in-gap states in a schematic (Fig. 5). In light of its complex electronic insight, PBDTTT-c appears plain and simple, as far as doped-state dynamics are concerned. This is in contrast to its complicated molecular design consisting of two heterocycles and extensive side-chain branches. In PBDTTT-c, we observe classic 1D-delocalization in agreement with the spectral response. The absorption line shape fits a textbook example for 1D Froehlich polaron excitations with relatively high coupling strength and higher effective hole mass. We fit the spectra using two generic oscillators, (Fig. 4, right) and find a proper match with the doped-state spectral features. The latter is not possible in rr-P3HT: P_2_ transitions are weaker despite more intense P_1_. We conclude that we have simply more polaronic states, a weaker Froelich coupling constant, thus less 1D-character. These conclusions are furthermore backed by the ground state spectra: In PBDTTT-c dispersion effects are minor as compared to rr-P3HT seen in the qualitative match of *ε*_2_ and the absorption coefficient *α*. In summary, we show that PBDTTT-c fits the classic description of a 1D Froelich polaron, while rr-P3HT has more complex spectral responses. This structural insight backs the concept of a 1D-polymers hence 1D-delocalization enhanced by push-pull systems. We conclude it is the inferior molecular symmetry of PBDTTT-c, which features suppressed *π*-stacking and thus translates to a plain doped-state electronic structure. This work aims to visualize the transition from pristine form to a doped-state for PBDTTT-c as part of a prominent family of push-pull type IR-active polymers for high-performance polymer photovoltaic cells. In direct comparison to rr-P3HT having a simpler core unit and stronger tendency to crystallize, PBDTTT-c offers suppressed *π*-stacking due to its complex structural asymmetry. In first view we find correlative spectral responses - both systems show intense polaron transitions and IRAV bands. The detailed scans, however, reveal the distinct difference, which highlights the simplicity of the electronic structure of PBDTTT-c. The systems resembles a similar response function in the doped state, as found for original conjugated polymers following the concept of 1D-delocalization. This insight points at the impact of molecular design, which can conserve a major polymer fingerprint - a 1D-character packed in an advanced, sophisticated chemical structure. In light of the high performance response from PBDTTT-c family in organic photovoltaics, our study supports the direction to assemble molecular designs, that foster a 1D-character.

## Methods

PBDTTT-c was purchased from Solarmer Inc., and used as received. P3HT bought from Rieke Metals (98%) and has been purified prior to use by re-crystallization in n-hexane. As electron acceptor the soluble fullerene derivative, (6,6)-phenyl-C61-butyric acid methyl ester (PCBM) was used. PCBM was purchased from Solenne Inc. (Groningen, The Netherlands). PBDTTT-c or P3HT and PBDTTT-c:PCBM or P3HT:PCBM (1:1 weight ratio) were dissolved in chlorobenzene with concentration of 10 g L^−1^ and 20 g L^−1^, respectively.

To measure the optical properties of the pristine and doped materials, NIR-Vis-UV variable angle spectroscopic ellipsometry (VASE) is used. The characterization is performed using a Woollam M-2000 (rotating compensator) ellipsometer (spectral range 0.73 to 6.5 eV). VASE results are analysed using WVASE software on basis of three phase layer model. The polymer film dielectric function is fitted using a parametric dispersion model based on generic oscillators. The layer thickness is measure beforehand by DekTak (Bruker). The as-derived preliminary optical model is consequently refined by point-to-point fits and the resulting *ε*_1_- and *ε*_2_-functions. The results are crosschecked in terms of the Kramers-Kronig consistency^41^,^42^. All VASE measurements are performed on polymer layers deposited by spin coating of the polymer solution on a glass substrate. Doped-state *in-situ* spectra are generated by exposing the sample to iodine vapor - after a saturation time (10 min) numerous VASE-spectra are recorded subsequently^31^.

The mid-IR spectra are recorded using a FTIR spectrometer (Bruker IFS66S) in attenuated total reflection (ATR) mode. A ZnSe crystal is used as the reflection element. The setup used for *in-situ* probing is presented in the supplementary part. Doped-state spectra are recorded in presence of an iodine crystal, which is placed in a closed volume box on top of the polymer-film and ATR-FTIR spectra are recorded *in-situ*. The plots relate the ground state signal (T_*ref*_, T is transmission) and subsequent doped-state spectra Ts to a differential spectra (−Δ*T*/*T*). We denote that (−Δ*T*/*T*) is absorbance A, in first approximation. For quantitative evaluations, we consider the intensity of transmission T as product of all 6 reflections (T equals R^6^). We can calculate intensities including Fresnel reflection coefficients assuming an isotropic material and the single attenuated total reflection from ZnSe-parallelepiped at 45° total reflection. In ATR-mode also photo-induced absorption is measured. We apply a lock-in mode, amplified by the FTIR spectrometer and a mechanical shutter. The polymer-fullerene blend is deposited on a ZnSe crystal and photo-excited at 532 nm (P3HT) and 664 nm (PBDTTT-c) diode-lasers thought the shutter. A differential spectra (−Δ*T*/*T*, T_*dark*_ and T_*light*_) are calculated from sequences of 1000 repetitions of recording 10 single beam spectra in the dark and light, respectively, to reduce the noise level. We denote that photo-induced VASE is not accessible with our equipment to date.

To evaluate the ATR-FTIR spectra for a quantitative analysis we relate the intensities of polarons and IRAVs to the electron-phonon coupling. The Froehlich interaction turns out to be suitable for polymers: The basis of the model is given by following Hamiltonian^41^:


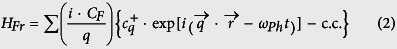


where the prefactor *C*_*F*_ is given by


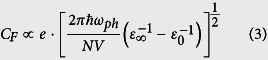


In those equations 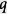 is a phonon wave vector, 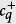 and c.c. are creation and annihilation operators, 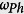 is a phonon frequency, 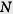 is the number of charges and 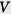 is a volume. The values of 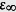 and 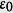 are high- and low-frequency dielectric constants. In this picture, electron phonon coupling is either enhanced, if the phonon frequency 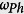 increases, or when the ‘‘static’’ dielectric constant 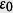 (below the energy of a selected vibronic transition) is strongly different from the dielectric constant 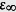 (above the energy of a selected vibronic transition), or equivalently,


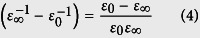


decreases. Our assumption is now based on the substitution of the phonon frequency by vibrational frequencies - in particular the IRAVs, which appear due to symmetry breaking (polymer deformation). Each one contributes to the difference (*ε*_0_ − *ε*_∞_), as well as in the strength of the polaron transition. For the case of chemical doping of rr-P3HT it is clear that the static dipole moment *ε*_0_ increases, which means that 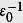 vanishes and the coupling coefficient increases in the Fröhlich model - thus allowing a quantitative interpretation.

## Additional Information

**How to cite this article**: Cobet, C. *et al*. Influence of molecular designs on polaronic and vibrational transitions in a conjugated push-pull copolymer. *Sci. Rep.* **6**, 35096; doi: 10.1038/srep35096 (2016).

## Supplementary Material

Supplementary Information

## Figures and Tables

**Table 1:** Summary of all measured optical transitions referring to the peak maxima (all units in eV).

Polymer	absorption edge	*π*-π*	*π*-*π*-stacking	P_1_	P_2_
rr-P3HT	1.90	2.30	2.10	1.50	0.35
PBDTTT-c	1.55	1.75	1.90	1.20	0.39

We denote the broadened shape of polaronic transitions.
