# Supplementary material for: Influence of molecular designs on polaronic and vibrational transitions in a conjugated push-pull copolymer
Source: Sci Rep. 2016 Oct 12;6:35096. doi: 10.1038/srep35096 (PMC5059657; doi:10.1038/srep35096)
Supplement: Supplementary Information [file srep35096-s1.pdf]

Influence of molecular designs on polaronic and vibrational transitions in a conjugated push-pull copolymer

Christoph Cobet[1,+], Jacek Gasiorowski[2,+], Reghu Menon[3], Kurt Hingerl[1], Stefanie Schlager[4], Matthew S. White[5], Helmut Neugebauer[4], N. Serdar Sariciftci[4], and Philipp Stadler[4,*]

[1]Center for Surface- and Nanoanalytics, Johannes Kepler University of Linz, A-4040 Linz, Austria

[2]Physics Department, Technical University of Chemnitz, 09107 Chemnitz, Germany

[3]Department of Physics, Indian Institute of Science, Bangalore 560012, India

[4]Institute of Physical Chemistry, Johannes Kepler University Linz, Altenbergerstr. 69, 4040 Linz, Austria

[5]Department of Physics, University of Vermont, Cook Building, 82 University Place, University of Vermont Burlington, VT 05405-0125, USA

*philipp.stadler@jku.at

+these authors contributed equally to this work

Supplementary Information

According to discussion on the relation of intensities of polaronic and IRAV peaks, for P3HT it suggests intense electron-phonon coupling. In addition to a number of theories describing IRAV behavior, we use additionally as a model picture, theory established for crystalline semiconductors. In theory the coupling between the charge and the phonon can be described by Fröhlich interaction, with an Hamiltonian[1]

$$H = \frac{\boldsymbol{p}^2}{2m_c} + \sum_{\boldsymbol{k}} \hbar\omega_{Ph} c_{\boldsymbol{k}}^{+} c_{\boldsymbol{k}} + \sum_{\boldsymbol{k}} \left(V_{\boldsymbol{k}} c_{\boldsymbol{k}} e^{i\boldsymbol{k}\cdot\boldsymbol{r}} + V_{\boldsymbol{k}}^{*} c_{\boldsymbol{k}}^{+} e^{-i\boldsymbol{k}\cdot\boldsymbol{r}} \right) \qquad (1)$$

In this equation, **r** describe the position of the operator of the charge with band mass m_c and momentum operator **p**. The $c_{\boldsymbol{k}}$ *and* $c_{\boldsymbol{k}}^{+}$ describe creation and annihilation operators of the longitudinal optical phonons with wave vector **k** and energy $\hbar\omega_{Ph}$. In this equation the electron-phonon coupling ($V_{\mathbf{k}}$) is described for inorganic materials in the arbitrary space dimensions as[1]

$$|V_{\boldsymbol{k}}|^2 = \Gamma\left(\frac{n-1}{2}\right) \frac{2^{\frac{n-3}{2}} \pi^{\frac{1}{2}(n-1)}}{V} \frac{\alpha}{k^{n-1}} \qquad (2)$$

Where $V_\mathbf{k}$ describes the volume (surface area) of a n-dimentional crystal, $\Gamma(x)$ is the Γ function described in details elsewhere and α the dimensionless factor describing the strength of the electron-phonon coupling and it is described as:

$$\alpha = \frac{e^2}{\hbar}\sqrt{\frac{m_c}{2\hbar\omega_{ph}}}\left(\frac{1}{\varepsilon_\infty} - \frac{1}{\varepsilon_0}\right) \qquad (3)$$

In this picture, electron phonon coupling is either enhanced, if the phonon frequency ω_{Ph} increases, or when the "static" dielectric constant ε_0 (below the energy of a selected vibronic transition) is strongly different from the dielectric constant ε_∞ (above the energy of a selected vibronic transition), or equivalently, $\left(\varepsilon_\infty^{-1} - \varepsilon_0^{-1}\right) = \frac{\varepsilon_0 - \varepsilon_\infty}{\varepsilon_0 \varepsilon_\infty}$ increases (as pointed out in the main text). These values can be found in from the dielectric function obtained to describe the optical absorption of polaron in the reflection spectroscopy. Using equation (3) we can also derive a general equation for a 1dimensional (1D) polaron describing the ground energy according to Peeters et al.[1]:

$$E_{1D}(\alpha) = \frac{n}{3} E_{3D}(A_1\alpha)$$

the polaron mass:

$$\frac{m_{1D}^*(\alpha)}{(m_c)_{3D}} = \frac{m_{3D}^*(A_1\alpha)}{(m_c)_{3D}}$$

the impedance function

$$Z_{1D}(\alpha) = Z_{3D}(A_1\alpha)$$

and polaron mobility

$$\mu_{1D}(\alpha) = \mu_{3D}(A_1\alpha).$$

with the product $(A_1\alpha)$ (proportionality factor for a 1D-system and strength of electron-phonon coupling). In the case of polymer each oscillator describes a specific polaron. As discussed for P3HT we found 3 oscillators, but only 2 for PBDTTT-c.

1. Peeters, F. M., Xiaoguang, W. & Devreese, J. T. Ground-state energy of a polaron in n dimensions. *Phys. Rev. B* **33,** 3926–3934 (1986).
